# Laparoscopic Complete Excision of the Posterior Muscular Cuff: Technique Refinements and Comparison With Stepwise Gradient Muscular Cuff Cutting for Hirschsprung Disease

**DOI:** 10.3389/fped.2022.578843

**Published:** 2022-04-05

**Authors:** Zebing Zheng, Zhu Jin, Mingjuan Gao, Chengyan Tang, Lu Huang, Yuan Gong, Yuanmei Liu

**Affiliations:** Department of Pediatric, General Thoracic and Urinary Surgery, Affiliated Hospital of Zunyi Medical University, Zunyi, China

**Keywords:** Hirschsprung disease, modified Soave, muscular cuff, enterocolitis, soiling, laparoscopy

## Abstract

**Objectives:**

Our institution had modified the Soave pull-through procedure using laparoscopic stepwise gradient muscular cuff cutting (LSGC) for Hirschsprung disease (HSCR). However, we found that a few children still suffered from obstructive symptoms and enterocolitis during the follow-up. Previous studies suggested that these symptoms might be caused by the retained muscular cuff. The purpose of this study was to employ a modified procedure of laparoscopic complete excision of the posterior muscular cuff (LCEPC) for HSCR and compare it with the laparoscopic stepwise gradient cutting muscular cuff (LSGC) procedure.

**Methods:**

Our institution records of 83 patients with classic form HSCR who underwent LSGC or LCEPC between August 2014 and July 2018 at the Pediatric Surgery Department of Zunyi Medical University (Zunyi, China) were carefully reviewed (LSGC, *n* = 52; LCEPC, *n* = 31). In the present study, we compared the postoperative complications and defecation functions of the two groups. All patients were followed-up (1–5 years, with an average of 2 years).

**Results:**

There were no differences regarding the operation time and the length of hospitalization between groups, while the anal dissection time in the LCEPC group (22.4 ± 4.8 min) was shorter than that of the LSGC group (45.5 ± 7.5 min) (*p* < 0.001). The postoperative complication of soiling was significantly increased in six patients (19.4%) in the LCEPC group compared with two patients (3.8%) in the LSGC group (*p* = 0.021). However, the total incidence of enterocolitis (two patients, 6.5%) was significantly decreased in the LCEPC group compared with the LSGC group (12 patients, 23.1 %) (*p* = 0.050). For anastomotic stricture, muscular cuff infection, and constipation, there were no significant differences between the two groups. No patients experienced bladder paralysis and incontinence postoperatively in this study. Anorectal manometries presented that the anorectal resting pressure was significantly lower in the LCEPC group (14.8 ± 2.7 mmHg) than the LSGC group (22.0 ± 3.8 mmHg), (*p* < 0.001).

**Conclusion:**

The laparoscopic complete excision of the posterior muscular cuff method was demonstrated as safe and efficient, with a decrease in the incidence of enterocolitis, although it may increase the number of soiling incidents in the short period post-surgery owing to a dissected partial internal anal sphincter.

## Introduction

Soave's first report on the endorectal pull-through without anastomosis approach to the treatment of Hirschsprung disease (HSCR) dates back to 1963 (1). With the rapid development of laparoscopic operations in the early 1990s, Georgeson et al. (2) reported a technique utilizing laparoscopic dissection of the rectum combined with anal mucosal dissection in 1995. Subsequently, many laparoscopic approaches to modified Soave–Georgeson procedures were described, including short muscular cuff anastomosis (3), long cuff dissection, and short V-shaped partially resected cuff anastomosis (4). A common complication was found in the literatures about the modified Soave–Georgeson procedure. These patients often had recurrent obstructive symptoms. Clinical features were presented as recurrent enterocolitis, constipation, and overflow incontinence (5). The purpose of these modifications was to decrease the postoperative complications due to internal anal sphincter achalasia and rectal cuff.

**Figure 1:**
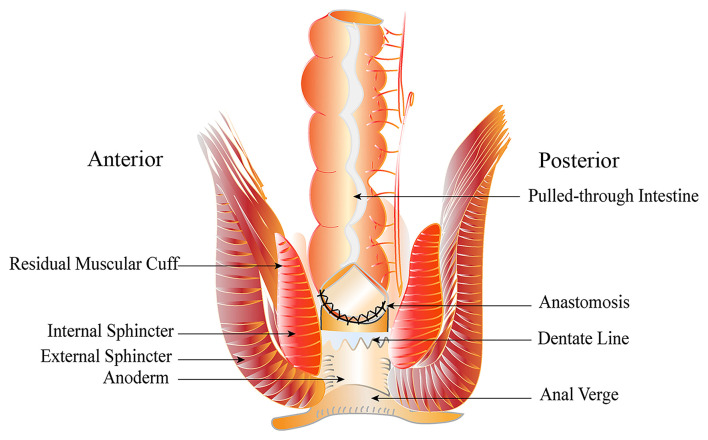
Diagram of laparoscopic stepwise gradient cutting muscular cuff (LSGC) procedure.

The laparoscopic Soave–Georgeson procedure was designed to protect the vital nerve and blood vessels of the pelvis from injury, by performing laparoscopic rectal dissection combined with endorectal dissection, and subsequently transanal pull-through. In the original descriptions, the submucosal dissection was extended above the peritoneal reflection at about 5–6 cm (6). However, the long muscular cuff could create a tight constricting band around the pulled-through bowel, which might increase the incidence of obstructive symptoms and enterocolitis. Amin et al. (7) used a short cuff operation that retained a muscular cuff of 1–2 cm and achieved excellent outcomes. Due to our increased experience to Soave–Georgeson operation, we have modified the Soave–Georgeson procedure that developed laparoscopic stepwise gradient cutting muscular cuff procedure and shortened the muscular cuff to ~1–2 cm in neonates and infants or 3–4 cm in children. Good results using the laparoscopic stepwise gradient cutting muscular cuff (LSGC) procedure (Figure 1) have been reported by Zheng et al. (8).

Although a few patients suffered from enterocolitis of the LSGC procedure, we found that the incidence of enterocolitis in patients with a 1–2 cm muscular cuff was lower than that in patients with a 3–4 cm muscular cuff. According to the above finding, we developed the laparoscopic complete excision of the posterior muscular cuff (LCEPC) procedure in July 2017. This study reports our institution's experience with two modified Soave methods for the purpose of developing an effective treatment technique for the classic form of Hirschsprung disease.

## Materials and Methods

### Patients

The medical records of 97 children admitted to the institution between August 2014 and July 2018 with a diagnosis of classical segment HSCR when the aganglionic segment did not extend beyond the upper sigmoid were carefully reviewed. Fourteen patients were excluded because of lost follow-up. Consequently, 83 patients were retrospectively reviewed in this study (male/female, 56:27; age, 2 months−6 years, with an average of 0.78 years). All patients were diagnosed with HSCR by barium enema, anorectal manometries, and biopsy pathologies. Ultimately, 52 patients received the LSGC operations, and 31 patients underwent LCEPC operations. The data collected included the operative time, anal dissection time, length of hospitalization, postoperative complications, and episodes of postoperative enterocolitis. A Stooling Survey was completed through a telephone interview or through scoring obtained from outpatient visit notes. Stooling scores comprised of a composite evaluation of stooling pattern (e.g., excessively loose, or explosive stooling), continence, and/or evidence of enterocolitis. The enterocolitis severity was graded using a previously designed scoring system ranging from grade I to grade III (9). The study protocols and their informed consents were reviewed and approved by the Medical Ethics Committee of Zunyi Medical University (approval no. ICUC-2014081934; Guizhou, China).

### Operative Technique

**Figure 2:**
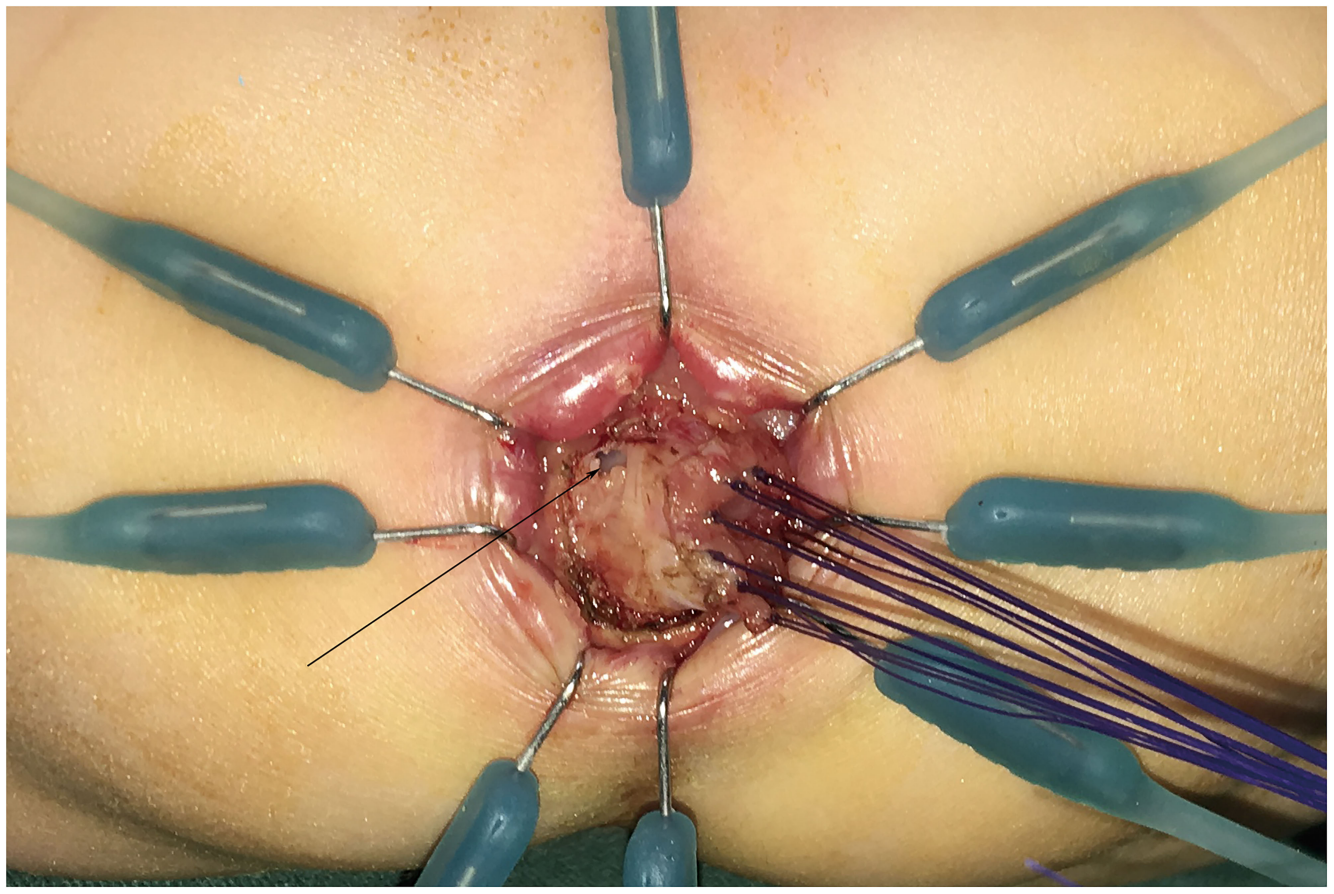
The rectal mucosa was separated through the anus for 1–2 cm to reach the rectal plane under laparoscopy (as the black arrow).

**Figure 3:**
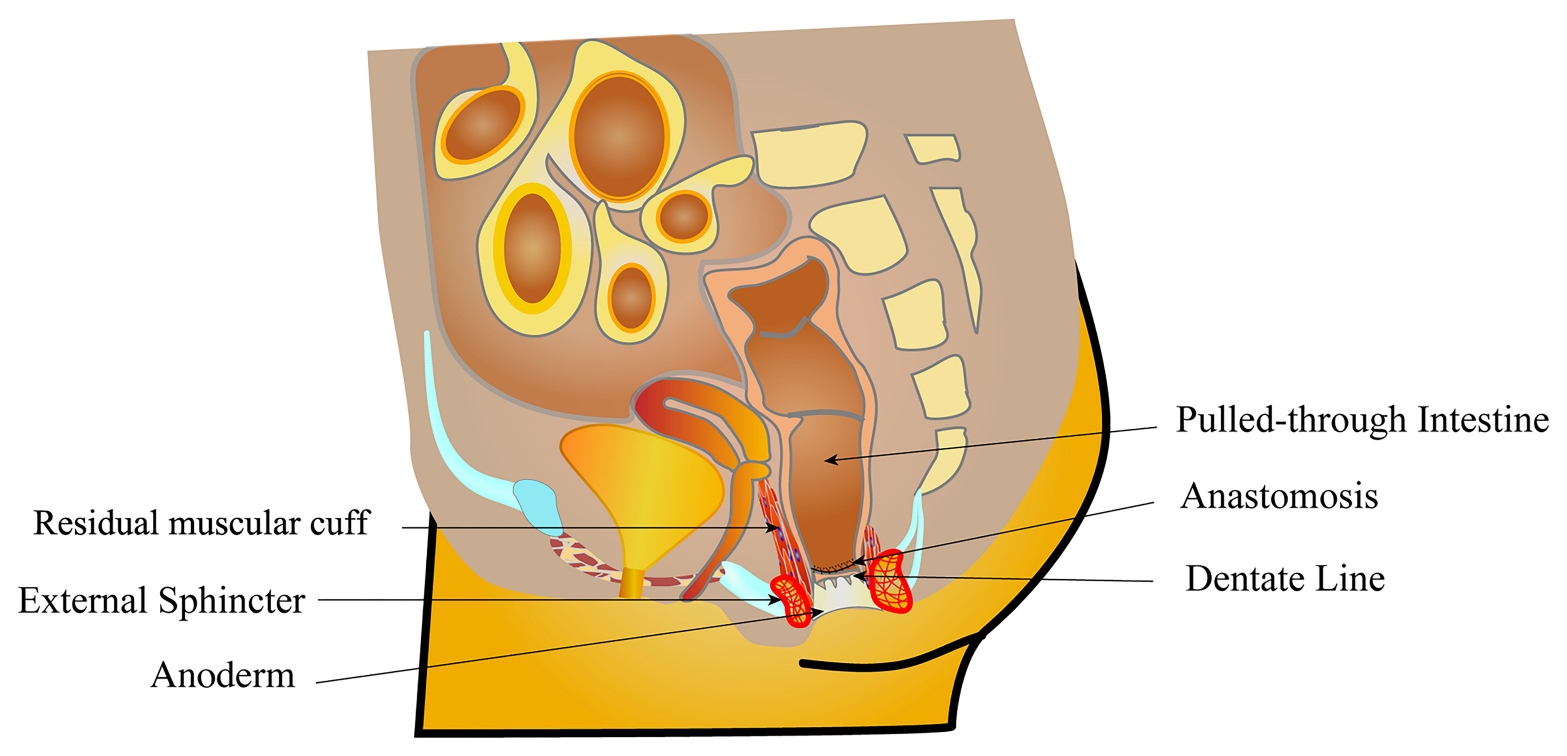
The muscle sheath 1–2 cm from the anterior wall of rectum was retained between the posterior wall of vagina and the pull-through colon. The muscular cuff of the posterior rectal wall was completely removed.

**Figure 4:**
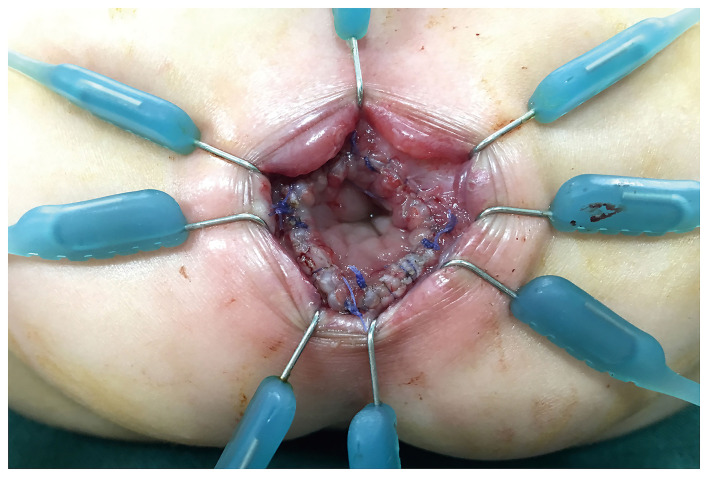
Completed anastomosis.

After general anesthesia induction, all patients were placed in a supine position perpendicular to the operating table with legs suspended from the body. A catheter was placed to decompress the bladder. The LCEPC procedure was performed for patients with HSCR confined to the classic segment based on a preoperative barium enema examination and acetylcholinesterase staining in a rectal suction biopsy. Typically, three ports are used with a 5-mm transumbilical camera trocar, and two 3- or 5-mm operative trocars at right and left lateral abdomen, respectively. At first, several intestinal myometrial biopsies were performed to detect the ganglion cells in the myometrial plexus. The aganglionic lengths were determined by biopsy and enema results. The mesentery of the colon was separated by laparoscopy with the vessel of the pull-through bowel preserved. Under the rectal peritoneal reflection, close to the rectal wall, separated with the electric hook, the anterior wall of the rectum were separated to the bladder neck or the posterior wall of the vagina. The posterior wall of the rectum was separated down to 1 cm above the dentate line (Supplementary Video 1). Anal retractors were used around the anal verge to expose the anus. A 4/0 Vicryl suture was placed as a traction suture 0.5–1.0 cm above the dentate line. The perineal steps involved the dissection of the rectal mucosa and the subsequent pull-through of the colon. The transanal mucosal rectal dissection started with a mucosal incision performed 0.5–1 cm above the dentate line (Supplementary Video 2). The blunt approach was used to separate the mucosa under the visible identification of a submucosal plane. The mucosectomy proceeded proximally for 1–2 cm until the plane of the rectal dissection (performed laparoscopically) had been reached (Figure 2). At this point, the rectum “prolapsed” outside. The rectal muscular layer (“cuff”) was sectioned at its distal part, and it was reintroduced into the pelvis after the section of the anterior and posterior edges. This technique was inspired by Dickie et al. for the treatment of problematic Soave cuff in HSCR (5). The posterior wall of the muscular cuff was completely removed along the left and right side, accounting for two-thirds of the whole circular muscular cuff to 0.5 cm of the dentate line edge, to avoid damaging the sphincter (Figure 3; Supplementary Video 3). One-third of the anterior wall of the muscular cuff was retained because there were afferent nerves controlling urination and defecation in the anterior wall of the muscular cuff. The next step was to pull through the colon inside the position of the remaining rectal muscular cuff until the normally ganglionic region (where there the biopsy was performed) was identified. The aganglionic bowel was resected, and Colo-anal anastomosis was performed (Figure 4).

The LSGC procedure was outlined by Zheng et al. (8) in a previous description. In brief, the aganglionic colon dissection by laparoscopy procedures was the same as the LCEPC procedure. The endorectal dissection was performed by a surgical incision of the stepwise gradient muscular cuff up toward the peritoneal cavity; the muscular cuff remained 1–2 cm in neonates and infants or 3–4 cm in children.

### Post-surgical Care

All patients were given intravenous broad-spectrum antibiotic (Ceftazidime for injection; 50 mg/kg body weight; GlaxoSmithKline S.p.A., Research Triangle Park, North Carolina, USA) therapy for 3–5 days, and an anal supporting tube was inserted and maintained for 3 days to prevent enterocolitis. Patients without a maintained nasogastric tube can be fed a liquid diet 6 h after the operation and normal milk 1 day after the operation. All patients were discharged about 7–8 days after surgery. Two weeks later, patients received anal dilatation based on the anal examination results. Anorectal resting pressure were conducted by anorectal manometries 6 months post-surgery.

### Statistical Analysis

Continuous data are expressed as mean ± standard deviation. A *t*-test was used to compare operative time, anal dissection, and the length of postoperative hospitalization between LSGC and LCEPC procedures. Qualitative data were presented as percentages and compared with the chi-squared test. Statistical analysis was carried out using SPSS19.0 (IBM SPSS Software, Armonk, NY, USA). A *p*-value of < 0.05 was considered statistically significant.

## Results

Between August 2014 and July 2018, 83 children with classic-form HSCR successfully underwent the two procedures and were routinely followed up. Patient demographics and clinical characteristics are presented in (Table 1). For patients in the LCEPC group, there had no significant difference in operative time (121.1 ± 9.7 min vs. 119.4 ± 13.7 min; *p* = 0.544) or length of hospitalization (8.9 ± 2.3 days vs. 9.9±3.1 days; *p* = 0.107) compared with the LSGC group. However, the anal dissection time in the LCEPC group (22.4±4.8 min) was shorter than that of the LSGC group (45.5 ± 7.5 min) (*p* < 0.001).

**Table 1:** Clinical outcomes in HSCR children undergoing surgery repair.

**Characteristic**	**Total**	**LCEPC**	**LSGC**	***p*** **-value**
**Postoperative course**				
Operative time (min) (n/mean ± SD)	83/120 ± 12.4	31/121.1 ± 9.7	52/119.4 ± 13.7	0.544^a^
Anal dissection time (min) (n/ mean ± SD)	83/36.8 ± 13.0	31/22.4 ± 4.8	52/45.5 ± 7.5	<0.001^a^
Length of hospitalization (day) (n/ mean ± SD)	83/9.5 ± 2.9	31/8.9 ± 2.3	52/9.9 ± 3.1	0.107^a^
**Postoperative complication (n/%)**				
Soiling	8/9.6	6/19.4	2/3.8	0.021^b^
Constipation	3/3.6	0/0	3/5.8	0.173^b^
Muscular cuff infection	4/4.8	0/0	4/7.7	0.113^b^
Anastomotic stricture	10/12	2/6.5	8/15.4	0.227^b^
Bladder paralysis	0/0	0/0	0/0	
Incontinence	0/0	0/0	0/0	
Enterocolitis	14/16.9	2/6.5	12/23.1	0.050^b^
Grade I	9/10.8	1/3.2	8/15.4	0.085^b^
Grade II	5/6.0	1/3.2	4/7.7	0.408^b^
Grade III	0/0	0/0	0/0	
Anorectal resting pressure (mmHg)	83/19.3 ± 4.9	31/14.8 ± 2.7	52/22.0 ± 3.8	<0.001^a^

*LCEPC, laparoscopic complete excision of the posterior muscular cuff; LSGC, laparoscopic stepwise gradient cutting muscular cuff*.

a *t-test*.

b *Chi-square test*.

The postoperative complications are listed in Table 1. The incidence of soiling in was significantly increased, with six patients (19.4%) in the LCEPC group compared with two patients (3.8%) in the LSGC group (*p* = 0.021). Regarding constipation, no patients in the LCEPC group reported such problems, but three patients (5.8%) in the LSGC group suffered from constipation; however, no significant difference was observed between groups (*p* = 0.173). One of three patients was diagnosed with the recurrence of HSCR, which was improved by LCEPC procedure. Regarding muscular cuff infection, no patients in the LCEPC group exhibited symptoms, but four patients (7.7%) in the LSGC group did; however, the difference was not significant (*p* = 0.113). Two patients (6.5%) in the LCEPC group and eight patients (15.4%) in the LSGC group exhibited anastomotic stricture, which was relieved by anal dilatation, but no significant difference was observed (*p* = 0.227). No incontinence and bladder paralysis were reported for either groups during long-term follow-up.

The total incidence of enterocolitis was significantly decreased with 2 patients (6.5%) in the LCEPC group compared to 12 patients (23.1%) in the LSGC group (*p* = 0.050). There was one patient (3.2%) diagnosed with grade I enterocolitis in the LCEPC group and eight patients (15.4%) in the LSGC group (*p* = 0.085). There was one patient (3.2 %) diagnosed with grade II enterocolitis in the LCEPC group and four patients (7.7 %) in the LSGC group (*p* = 0.408). No patient experienced grade III enterocolitis in either of the two groups. The severity of these episodes was not significantly different between the two groups. Anorectal manometries indicated that the anorectal resting pressure was significantly lower in the LCEPC group (14.8 ± 2.7 mmHg) than the LSGC group (22.0 ± 3.8 mmHg) (*p* < 0.001).

## Discussion

The Soave approach and endorectal dissection were designed to prevent injury to structures surrounding the rectal wall, especially the nerves of the bladder and sexual function management with HSCR (9). However, Swenson (10) reported that the Soave procedure left a long aganglionic muscular cuff, which might extrinsically obstruct the pull-through bowel and influence peristalsis on the normal colon. Many studies indicated that the long aganglionic muscular cuff may be related to the postoperative obstructive symptoms, constipation, and enterocolitis in HSCR patients (6, 11). Subsequently, other surgical procedures that shortened the length of the muscular cuff were developed to relieve the above complications (4). Wester et al. (12) recommend a residual 1–2 cm muscular cuff. Short muscular cuff reduced the incidence of enterocolitis to 17.5% and also shortened the hospital stay. Yang et al. (4) reported a long cuff dissection and a short V-shaped resected cuff anastomosis procedures that reduced the incidence of anastomotic stricture and constipation. Nevertheless, these short muscular cuff procedures may increase the risk of damage to perirectal nerves and anal sphincter.

In our center, we have carried out the stepwise gradient cutting muscular cuff procedure since 2003 and shortened the muscular cuff to avoid the long aganglionic muscular cuff problem (8). We found that the shortened muscular cuff procedures could decrease the incidence of enterocolitis and obstruction symptoms. However, the incidence of enterocolitis was still higher than some other studies reported, and there were some patients who suffered from constipation during the follow-up period (3). In 2013, Levitt et al. (13) reported a novel modification of Swenson's original transabdominal dissection concept using full-thickness rectal dissection for HSCR; all the patients achieved voluntary urinary and fecal continence after operations, and the incidence of enterocolitis was only 14%. We carefully reviewed the data described by Sherman et al. (14) and adopted the Swenson operation, and since then, there had no anastomosis obstructive problem post-surgery, and the rate of post-operative enterocolitis was much lower than other resection procedures.

Based on the understanding and experience of Soave operation and full-thickness rectal dissection from our institution and other famous clinical centers (13), we modified Soave as LCEPC by the following changes: the posterior wall of the muscular cuff was completely removed along the left and right sides, accounting for two-thirds of the whole circular muscular cuff to 0.5 cm of the dentate line edge, to avoid damaging the sphincter. One third of the anterior wall of the muscular cuff was retained. The modified Soave procedure with a complete excision of the posterior muscular cuff was similar to the modified Swenson procedure, although there had been some differences between the two procedures. First, in modified Swenson procedure, the rectum below the peritoneal reflection was dissociated up to the inferior border of the levator ani muscle. This process might damage the pelvic strictures, especially the pelvic nerves and the bladder/vagina. The higher anal anastomosis was performed if the rectum was not dissociated enough by laparoscopy, which increased the risk of anastomotic leakage and recurrence of HSCR. However, the transanal mucosal rectal dissection started with a mucosal incision. The mucosectomy proceeded proximally for 1–2 cm until the plane of the rectal dissection (performed laparoscopically) had been reached in our study, which protected the structure around the rectum. Second, the full-thickness rectal dissection was performed from the Herrmann line in modified Swenson procedure, which was higher than our study. Finally, the whole aganglionic muscular cuff was removed by circumferential full thickness in modified Swenson procedure. However, the muscular cuff of the anterior wall of the rectum was retained in our study, which was the essential nerves access position that controlled the bladder and sexual function.

Laparoscopic separation of the rectum in the pelvic cavity and transanal resection of the muscular sheath of the posterior wall of the rectum were important steps in the LCEPC. To decrease the time of anal dilatation and extension in transanal procedure, the rectal dissociation by laparoscopy is required as lower as possible (11). In the LCEPC procedure, under the rectal peritoneal reflection, the anterior wall of the rectum was easily separated from the bladder neck or the posterior wall of the vagina. The posterior wall of the rectum was separated down to 1 cm above the dentate line. Some doctors considered that too much separated rectum below the peritoneal reflection might increase the risk of injury to the pelvic nerves and bladder/vagina (15). Therefore, the long muscular cuff anastomosis was a highly praised procedure for HSCR. During our practice, the posterior wall of rectum had loose knot and hoof tissue, which was convenient for us to dissociate rectum with electric hook. However, excessive bleeding would obscure the surgical vision; hemostasis should be manipulated meticulously. The anal dissection time was shorter than LSGC procedures, which was partially owing to the dissociation of rectum by laparoscopy. No bladder paralysis or vaginal and urethral complications were presented in this study.

We observed 6.5% of enterocolitis in the LCEPC procedures. Enterocolitis is the major cause of morbidity and mortality in HSCR. However, the etiology of enterocolitis is multivariable and poorly understood, and the obstructive cuff alone is likely to participate in its development (16). Enterocolitis has a widely variable incidence of between 4.6% and 54%, and no sufficient evidence shows whether the incidence correlates with the type of residual muscular cuff performed (17). In our study, the relatively low rate of enterocolitis in the LCEPC procedures may be due to the complete removal of muscular cuff at the posterior wall of the rectum and the 1–2 cm muscular cuff retained at the anterior wall of the rectum. This was carried out to avoid obstructive symptoms and decrease the incidence of constipation and anastomotic stricture as shown in our patients. Studies reported that 75% patients with recurrent enterocolitis following a pull-through operation had improved symptoms after a sphincterotomy (18). Some surgeons would be worried about the postoperative incontinence as a result of sphincterotomy and potential permanent injury to the sphincter (19). During our clinical practice, preserving the partial internal anal sphincter below the dental line is the key to postoperative continence. At the same time, we retained 1–2 cm of muscular cuff between the pull-through and the bladder/vagina to reduce the potential damage to the bladder/vagina and nerves of sexual function. However, sexual function needs long-term follow-up.

Compared to the previous studies, the occurrence of soiling in the LSGC procedure group was decreased (3.8%). Soiling is another postoperative complication after pull-through for HSCR. Levitt et al. (20) described that continence is related to normal anal sensation, voluntary sphincter control, and appropriate colonic motility. Some researchers reported that the muscular cuff participated in part in the function of internal sphincter and maintained the control function of defecation (21). Interruption of any of these elements leads to HSCR children being partially or totally incontinent. However, the soiling is pseudoincontinent, implying that the continence mechanism is intact. Many studies have reported that soiling improved with the follow-up time (22). In this study, we found that 19.4% of soiling in the LCEPC procedure was higher than the LSGC procedure, and the anal resting pressure was significantly lower in the LCEPC group. Tran et al. (23) reported that the internal and external anal sphincters contributed to 55.0% and 35.0% of the anal resting pressure, respectively. Therefore, the decreased anal resting sphincter pressure was related to the partial resection of the internal anal sphincter in the LCEPC procedure. In our series, patients presented with occasional soiling after breaking wind. Patients were evaluated by contrast enema and an anal examination for the integrity of the anal canal. Medical management started for those who presented with soiling 2 weeks after pull-through. Laxatives were prescribed for patients with colonic dilation, loperamide, and a special dietary regimen (constipating diet). All patients' symptoms were gradually improved by medical management 6 months post-surgery. We advocate that the complete excision of posterior muscular cuff is beneficial to both the continence or the enterocolitis.

This study has some limitations; some of the limitations and drawbacks of this study are the small sample size and the short follow-up period, limiting its generalizability. Further studies in larger patient groups, multicenter study, and long-term follow-up are needed. Another is that this observational study has some degree of heterogeneity relating to several factors that the study was not randomized controlled and certified by multiple medical institutions, with a different surgeon.

In summary, the laparoscopic complete excision of the posterior Soave cuff procedure for classic segment HSCR demonstrated safety and efficacy, with a decrease in the incidence of enterocolitis.

## Data Availability Statement

The original contributions presented in the study are included in the article/Supplementary Material, further inquiries can be directed to the corresponding author.

## Ethics Statement

The studies involving human participants were reviewed and approved by Ethics Committee of Affiliated Hospital of Zunyi Medical University. Written informed consent to participate in this study was provided by the participants' legal guardian/next of kin. Written informed consent was obtained from the individual(s), and minor(s)' legal guardian/next of kin, for the publication of any potentially identifiable images or data included in this article.

## Author Contributions

ZZ performed the LCEPC procedure and collected data. ZJ performed the LCEPC procedure. MG, CT, LH, and YG analyzed the data. YL conceived and designed the experiments, performed the LSGC and LCEPC procedures, contributed reagents, materials, and analysis tools. All authors reviewed drafts of the article and approved the final draft.

## Funding

This work was supported by the Natural Science Foundation of China (NSFC 82060100 and NSFC 81650029), Fund of the Department of Guizhou Science and Technology of China (Nos. ZK2021361 and 20204Y005), and the School Fund of Zunyi Medical University (No. 201426).

## Conflict of Interest

The authors declare that the research was conducted in the absence of any commercial or financial relationships that could be construed as a potential conflict of interest.

## Publisher's Note

All claims expressed in this article are solely those of the authors and do not necessarily represent those of their affiliated organizations, or those of the publisher, the editors and the reviewers. Any product that may be evaluated in this article, or claim that may be made by its manufacturer, is not guaranteed or endorsed by the publisher.

## Abbreviations

HSCR: Hirschsprung disease
LSGC: laparoscopic stepwise gradient cutting muscular cuff
LCEPC: laparoscopic complete excision of the posterior muscular cuff.
